# Residual non-specific and disease-specific inflammatory markers in successfully treated young psoriasis patients: a cross-sectional study

**DOI:** 10.1007/s12026-024-09584-4

**Published:** 2025-01-08

**Authors:** Eva Klara Merzel Šabović, Tadeja Kraner Šumenjak, Mojca Božič Mijovski, Miodrag Janić

**Affiliations:** 1 Department of Dermatovenerology, University Medical Centre Ljubljana, Gradiškova ulica 10, Ljubljana, Slovenia; 2 Faculty of Medicine, University of Ljubljana, Vrazov trg 2, Ljubljana, Slovenia; 3 Faculty of Agriculture and Life Sciences, University of Maribor, Pivola 10, Hoče, Slovenia; 4 Department of Vascular Diseases, Laboratory for Haemostasis and Atherothrombosis, University Medical Centre Ljubljana, Zaloška 7, Ljubljana, Slovenia; 5 Department of Endocrinology, Diabetes and Metabolic Diseases, University Medical Centre Ljubljana, Zaloška 7, Ljubljana, Slovenia

**Keywords:** Psoriasis, Biologic therapy, Inflammation, Cytokines

## Abstract

Psoriasis is a chronic, immune-mediated disease. The systemic inflammation triggered by psoriasis contributes significantly to increased cardiovascular risk. While various treatments completely clear the skin, the associated effects on systemic inflammation are not yet clear. We investigated residual systemic inflammation in successfully treated patients. Circulating disease-specific and non-specific inflammatory markers were measured and compared in 80 psoriasis patients (aged 30–45 years) successfully treated with topical therapy, methotrexate, adalimumab, secukinumab or guselkumab, and in 20 healthy controls. Non-specific inflammatory markers (high-sensitivity C-reactive protein (hs-CRP), complete blood count (CBC) parameters, neutrophil-to-lymphocyte ratio (NLR), platelet-to-lymphocyte ratio (PLR), mean platelet volume-to-platelet ratio (MPR), and red blood cell distribution width-to-platelet ratio (RPR)) and disease-specific inflammatory markers (interferon-γ (IFN-γ), tumor necrosis factor (TNF), interleukin (IL)-1β, IL-12p70, IL-17, and IL-23) were measured and compared between groups. Disease-specific cytokines (IFN-γ, TNF, IL-1β, IL-12p70, and IL-17, but not IL-23), were significantly elevated in patients compared to controls, while non-specific inflammatory markers showed no differences compared to controls. The residual disease-specific cytokines were similarly elevated in all five treated groups. In addition, they correlated significantly with body mass index (BMI) and waist circumference. Our results suggest that psoriasis patients have elevated residual disease-specific cytokines despite successful treatment, while the non-specific inflammatory markers are similar to those in control subjects. Residual disease-specific inflammatory markers correlated with BMI and waist circumference. A possible beneficial effect of body weight control in psoriasis patients merits further investigation. The study was registered at http://clinicaltrials.gov (identifier: NCT05957120) on July 24, 2023.

## Introduction

Psoriasis is a chronic immune-mediated disease that affects 2–3% of the population and manifests itself in the form of psoriatic skin lesions [1]. In recent years, the pathophysiology of psoriasis has revealed a dysregulation of the immune system consisting of a complex interplay between different cytokines, in particular the Th1 and Th17 cytokine family [1, 2]. Systemic inflammation can be exacerbated by common comorbidities, especially metabolic disorders [3]. Inflammation contributes to the cardiovascular and metabolic risk associated with morbidity and mortality in psoriasis patients [4]. Currently, there are several effective treatment options for moderate to severe psoriasis, including biologic therapies that target disease-specific cytokines [5, 6]. As a result, successful treatment has become possible for most patients. However, it is not yet fully understood whether the resolution of skin lesions is also associated with a reduction or normalization of complex systemic inflammation.

Systemic inflammation can be measured indirectly by circulating inflammatory markers [7]. Acute phase reactants such as C-reactive protein (CRP), erythrocyte sedimentation rate, and fibrinogen, all non-specific markers of inflammation, have been shown to decrease following systemic treatment with methotrexate or biologic therapy [8]; less is known about disease-specific markers of inflammation. Psoriasis is characterized by the dermal and systemic secretion of certain pro-inflammatory cytokines of the Th1 and Th17 cytokine family, such as interferon-γ (IFN-γ), tumor necrosis factor (TNF), interleukin (IL)-6, IL-17, IL-22, and IL-23 [9]. This secretion can be exacerbated by concomitant diseases such as metabolic syndrome [3]. Various disease-specific circulating inflammatory markers have been shown to decrease after treatment [10, 11]. Overall, the exact data on the remaining non-specific and disease-specific inflammatory burden, especially in successfully treated psoriasis patients, has not yet been conclusively clarified. This is particularly true for patients treated with biologic therapies targeting specific cytokines.

The aim of the present study was to investigate whether non-specific and disease-specific inflammatory markers are still elevated in young patients with psoriasis who have been successfully treated compared to healthy controls of the same age. We hypothesized that complete clearance of skin lesions would also be associated with normalization of non-specific and disease-specific inflammatory markers, which have not been investigated before.

## Materials and methods

### Study population and design

We conducted a cross-sectional study of 80 patients (54 men and 26 women) with psoriasis vulgaris and 20 healthy controls, of the same age and sex (11 men and 9 women) at the Dermatology Outpatient Clinic, Department of Dermatovenerology, University Medical Centre Ljubljana, Ljubljana, Slovenia. We recruited consecutive patients who were effectively treated with topical therapy (*n* = 21), methotrexate (*n* = 11), adalimumab (*n* = 14), secukinumab (*n* = 14), or guselkumab (*n* = 20). These five treatments were selected because they are the most commonly used treatments for psoriasis in Slovenia. The last three are human monoclonal antibodies against interleukins, which are associated with the development of atherosclerosis and cardiovascular risk based on clinical studies or hypothetical mechanisms. These treatment groups also indirectly reflect the severity of psoriasis, with mild cases effectively treated with topical therapy, and moderate to severe cases treated with methotrexate or biologic therapy. Participant recruitment lasted from March 2022 to December 2023, and treatment efficacy was defined by the Psoriasis Area Severity Index (PASI). Treatment was rated as excellent (PASI < 5) in 99% of patients and good (PASI 5–7) in 1% of patients. Both patients and physicians had to be satisfied with the response to treatment and had to have no intention of changing the treatment. All participants met the following inclusion criteria: diagnosis of psoriasis; age between 30 and 45 years; effective treatment with topical therapy, methotrexate, adalimumab, secukinumab, or guselkumab; and stable clinical course in terms of PASI score for at least 6 months. We chose an age range of 30 to 45 years to avoid the effects of various diseases that affect older patients more frequently and that could affect inflammation and thus jeopardize our objective [35]. Exclusion criteria were previous cardiovascular events, type 1 or type 2 diabetes, menopause, pregnancy or breastfeeding, psoriatic arthritis or other chronic inflammatory diseases, and other treatments in addition to psoriasis treatment. Healthy control subjects aged 30 to 45 years were included with the same exclusion criteria. All participants took part in this study voluntarily and gave their written informed consent. The study is registered at http://clinicaltrials.gov (ClinicalTrials.gov Identifier: NCT05957120). The reporting of this study is in accordance with the STROBE guidelines [36]. The study was approved by the Slovenian National Medical Ethics Committee (approval number 0120–422/2021/6). All methods were performed in accordance with the Declaration of Helsinki of 1975 as amended in 2013.

### Study protocol

During the study appointment, a complete medical history was obtained from each study participant, and a complete medical examination was performed, including duration of psoriasis, duration of treatment, and smoking status. Each participant’s anthropometric measurements (weight, height, and waist circumference), systolic and diastolic blood pressure, and heart rate were determined. In addition, fasting blood samples were taken from each participant by venipuncture according to the standard procedure and collected in vacuum tubes. Two types of inflammatory markers were determined from the collected blood samples: (i) non-specific inflammatory markers (standard markers, high-sensitivity CRP (hs-CRP) and complete blood count (CBC) markers; novel CBC-derived inflammatory markers, neutrophil-to-lymphocyte ratio (NLR), platelet-to-lymphocyte ratio (PLR), median mean platelet volume-to-platelet ratio (MPR), and red blood cell distribution width-to-platelet ratio (RPR)) and (ii) selected disease-specific inflammatory markers, namely IFN-γ, TNF, IL-1β, IL-12p70, IL-17, and IL-23.

### Laboratory methods

A complete differential blood count was performed on the XN-1000 hematology analyzer (Sysmex, Japan). The serum was prepared by centrifugation at 2000 × g for 20 min. Immediately after centrifugation, serum was aliquoted and stored at − 70 °C until analysis. Serum levels of IFN-γ, TNF, IL-1β, IL-12p70, IL-17, and IL-23 were measured with xMAP© technology using magnetic beads coupled with specific antibodies (all R&D Systems, USA) on a MagPix instrument (Luminex Corporation, USA). We calculated the NLR, PLR, MPR, and RPR using the following equations: NLR = neutrophil count/lymphocyte count, PLR = platelet count/lymphocyte count, MPR = mean platelet volume/platelet count, and RPR = red cell distribution width/platelet count.

### Statistical analysis

Statistical analyses were carried out using the software R (version 4.2.2) and IBM SPSS Statistics 28. Due to the non-normal distribution of the data and the presence of outliers, patient characteristics were represented by group medians and interquartile ranges. The non-parametric Kruskal–Wallis test or the Mann–Whitney *U* test was used to test the null hypothesis that the population medians are all equal. If the null hypothesis was rejected, the Dunn multiple comparison test with Bonferroni correction was used to determine which specific median pairs showed significant differences. Structural percentages were reported for two categorical variables and Fisher’s exact test was also performed. To determine whether inflammatory markers varied by type of psoriasis treatment, Quade’s nonparametric ANCOVA test was used, with systolic blood pressure and age included as covariates in the model. In cases where ANCOVA yielded significant results, pairwise comparisons were performed using Fisher’s least significant difference (LSD) method. As this method does not sufficiently control the type I error rate, the Benjamini–Hochberg correction was then applied.

## Results

**Fig. 1:**
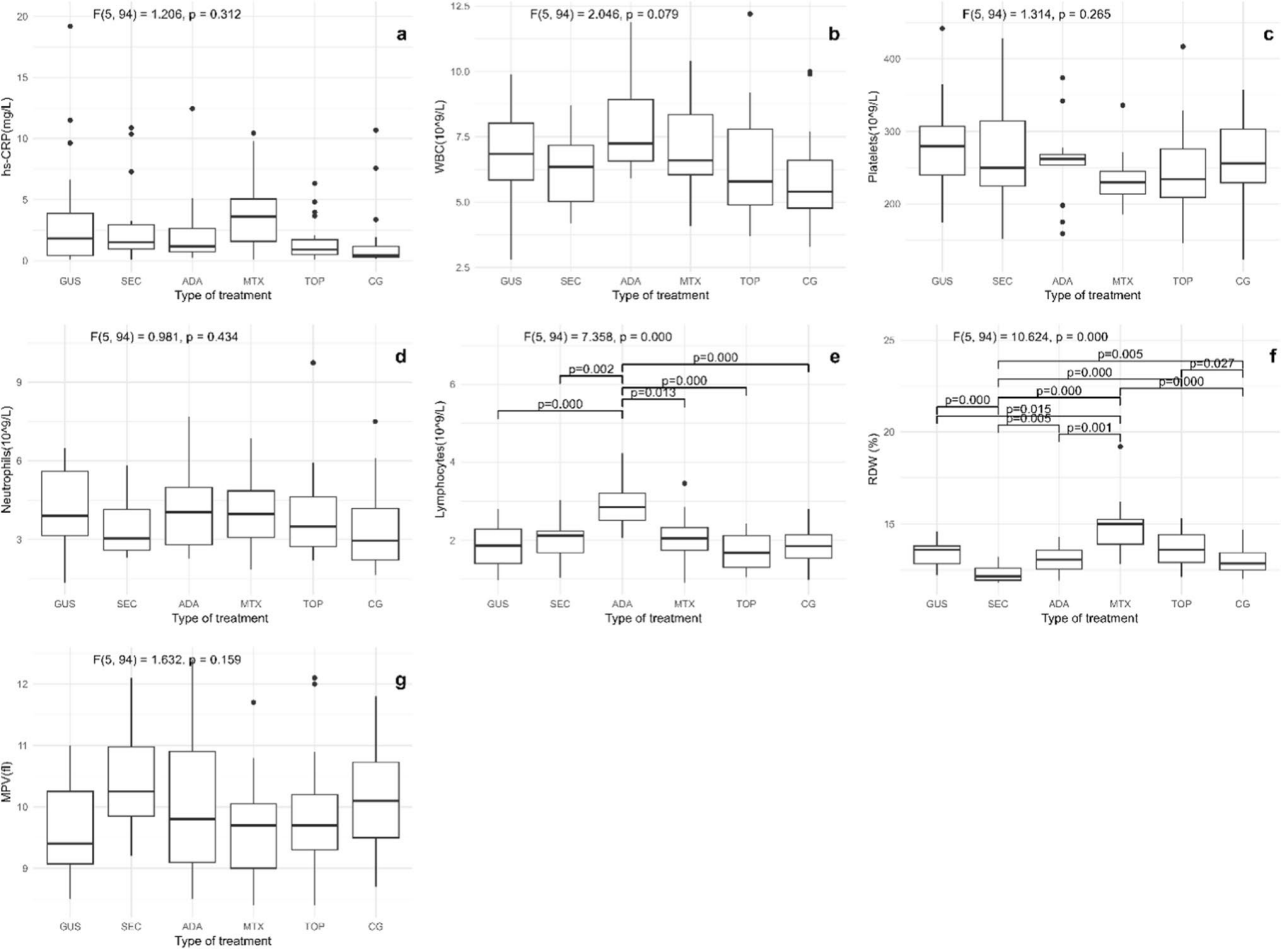
Non-specific inflammation markers in the five groups of patients with psoriasis and the control group. **a** hs-CRP, **b** WBC, **c** platelets, **d** neutrophils, **e** lymphocytes, **f** RDW, and **g** MPV. Hs-CRP, high-sensitivity C-reactive protein; WBC, white blood cells; RDW, red cell distribution width; MPV, mean platelet volume; GUS, guselkumab; SEC, secukinumab; ADA, adalimumab; MTX, methotrexate; TOP, topical therapy; CG, control group. Quade’s ANCOVA and LSD post hoc test with Benjamin–Hochberg correction was applied, with only statistically significant pairs shown in the figure (*p* ≤ 0.05)

**Fig. 2:**
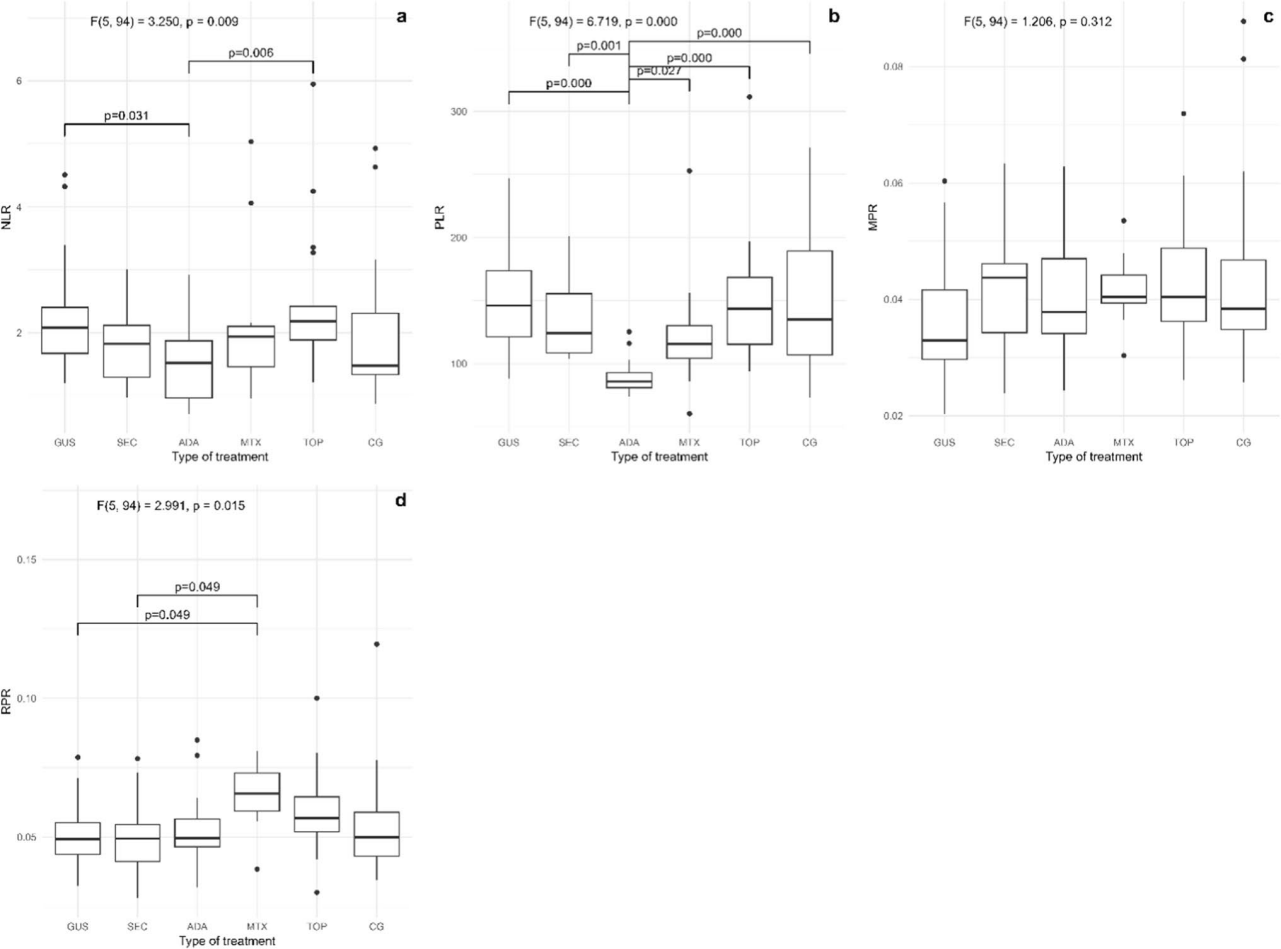
CBC-derived non-specific inflammation markers in the five groups of patients with psoriasis and the control group. **a** NLR, **b** PLR, **c** MPR, and **d** RPR. CBC, complete blood count; NLR, neutrophil-to-lymphocyte ratio; PLR, platelet-to-lymphocyte ratio; MPR, median mean platelet volume-to-platelet ratio; RPR, mean red cell distribution width-to-platelet ratio; GUS, guselkumab; SEC, secukinumab; ADA, adalimumab; MTX, methotrexate; TOP, topical therapy; CG, control group. Quade’s ANCOVA and LSD post hoc test with Benjamin–Hochberg correction was applied, with only statistically significant pairs shown in the figure (*p* ≤ 0.05)

**Fig. 3:**
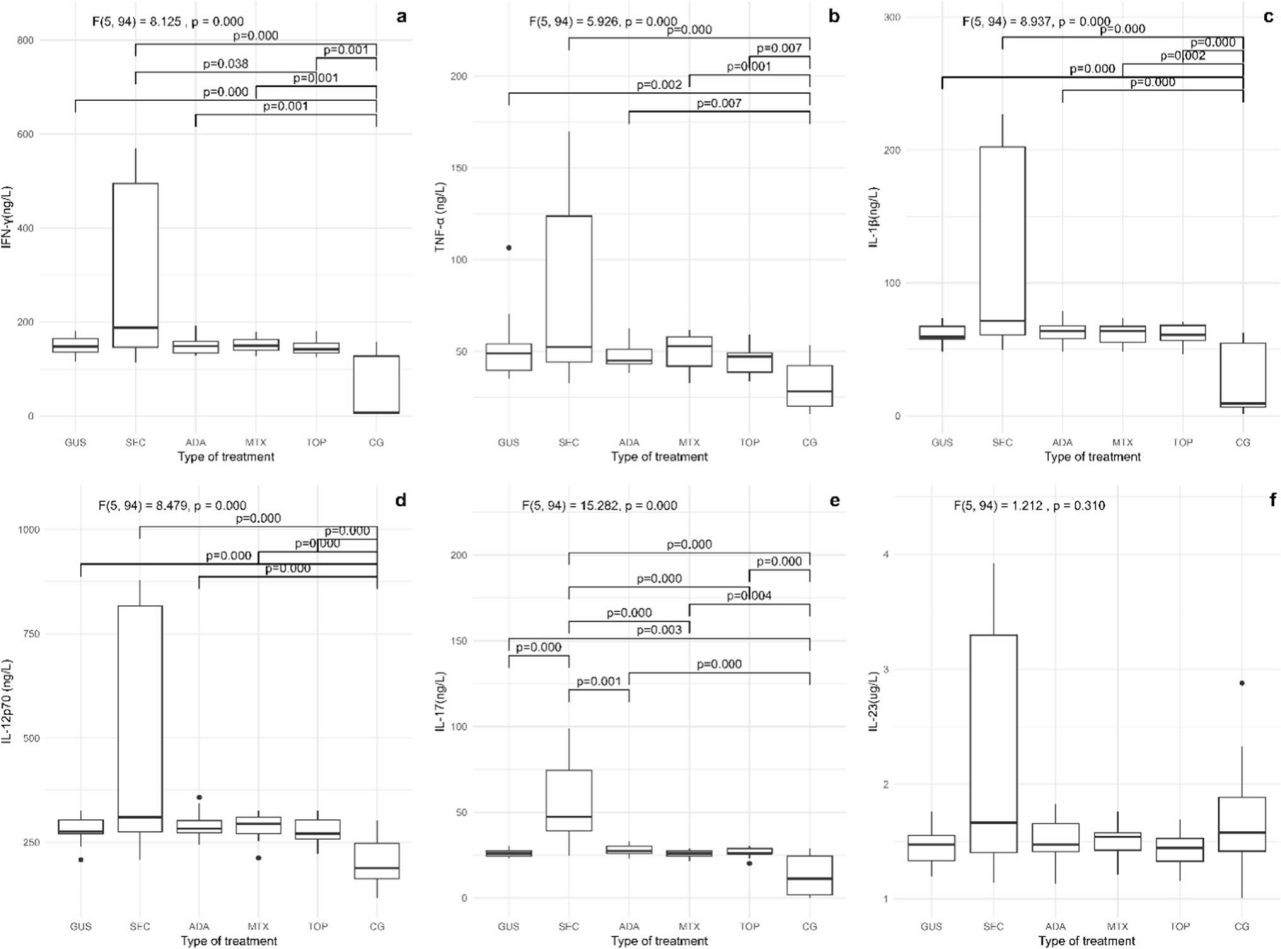
Disease-specific inflammation markers in the 5 groups of psoriasis patients and the control group. **a** IFN-γ, **b** TNF, **c** IL-1β, **d** IL-12p70, **e** IL-17, and **f** IL-23. IFN-γ, interferon-γ; TNF, tumor necrosis factor; IL, interleukin; GUS, guselkumab; SEC, secukinumab; ADA, adalimumab; MTX, methotrexate; TOP, topical therapy; CG, control group. Quade’s ANCOVA and LSD post hoc test with Benjamin–Hochberg correction was applied, with only statistically significant pairs shown in the figure (*p* ≤ 0.05)

The characteristics of the patients are listed in Table 1. Of the included patients, 37% were smokers and 65% had an elevated body mass index (BMI) and waist circumference, while in the control group, 30% were smokers and 40% had an elevated BMI and waist circumference. We examined three groups of inflammatory markers: two groups of non-specific inflammatory markers (standard inflammatory markers (hs-CRP and CBC markers), shown in Fig. 1 and novel CBC-derived systemic inflammatory biomarkers (NLR, PLR, MPR, and RPR), shown in Fig. 2) and selected disease-specific inflammatory markers (IFN-γ, TNF, IL-1β, IL-12p70, IL-17, and IL-23), shown in Fig. 3. There were no consistent, generalized significant differences in the non-specific inflammatory markers, i.e., hs-CRP and the parameters of CBC (white blood cells, platelets, neutrophils, lymphocytes, red blood cell distribution width (RDW), and mean platelet volume (MPV)) compared to controls, as shown in Fig. 1. Values of hs-CRP did not differ between patients and controls, and no other marker differed between the entire patient group and controls. Of the seven parameters measured, significant differences were found for only two (lymphocytes and RDW)—the adalimumab group had a higher lymphocyte count than all other groups and the methotrexate group had a higher RDW than all other groups (*p* < 0.05). In addition, no consistent, overall differences were observed in novel CBC-derived non-specific markers reflecting systemic inflammation. Of the four parameters measured (NLR, PLR, MPR, and RPR), there was only a significant difference (*p* < 0.05) in NLR, which was lower in the adalimumab group compared with the topical group and in the guselkumab group, and in PLR, which was lowest in adalimumab compared with all other groups (Fig. 2).

**Table 1:** Characteristics of patients

**Patients’ characteristics**	**CG** (*n* = 20)	**TOP** (*n* = 21)	**MTX** (*n* = 11)	**ADA** (*n* = 14)	**SEC** (*n* = 14)	**GUS** (*n* = 20)	**Test statistics *p*-value**
Average age (years)	34.50 (31.25–39.75)	38.00 (32.00–41.50)	39.00 (35.00–42.00)	39.50 (36.75–41.00)	39.50 (34.50–43.25)	40.00 (36.00–43.00)	*H* = 9.312 *p* = 0.097
Sex							
Male	12	13	7	10	11	13	Exact test *p* = 0.905
Female	8	8	4	4	3	7	
Smokers no. (%)	6 (30)	7 (33)	3 (27)	7 (50)	8 (57)	4 (20)	Exact test *p* = 0.242
Duration of psoriasis (years)	/	8.0 (4.5–20.0)	10.0 (5.0–12.0)	20.0 (11.5–25.0)	16.5 (13.75–23.25)	20.0 (15.0–23.5)	*H* = 16.646 *p* = 0.002
Duration of treatment (months)	/	77.0 (47.0–239.0)	29.0 (21.0–47.0)	95.0 (61.0–117.5)	48.0 (33.5–59.0)	31.0 (27.3–51.0)	*H* = 31.059 *p* < 0.001
BMI (kg/m^2^)	24.30 (23.40–26.75)	23.36 (22.59–26.18)	28.05 (22.65–37.51)	27.04 (23.62–30.66)	31.04 (26.75–35.39)	27.50 (24.54–34.96)	*H* = 17.766 *p* = 0.003
Waist circumference (cm)	90.00 (79.75–94.00)	86.00 (77.50–94.50)	104.00 (92.00–121.50)	93.25 (89.125–109.375)	104.50 (98.00–113.50)	99.25 (85.625–108.875)	*H* = 24.767 *P* < 0.001
PASI	/	0.2 (0.1–1.75)	1.2 (0.7–3.2)	0.0 (0.0–0.2)	0.0 (0.0–0.65)	0.0 (0.0–0.6)	*H* = 17.393 *p* = 0.002
BSA (m^2^)	/	1.0 (1.0–1.5)	2.0 (1.0–4.0)	0.0 (0.0–0.25)	0.0 (0.0–1.0)	0.0 (0.0–1.0)	*H* = 20.849 *p* < 0.001
Systolic BP (mmHg)	127.00 (116.00–138.00)	124.00 (110.00–132.50)	137.00 (129.00–142.00)	121.00 (110.25–133.25)	123.00 (118.00–133.25)	120.00 (110.75–136.75)	*H* = 8.948 *p* = 0.111
Diastolic BP (mmHg)	84.50 (77.25–90.00)	79.00 (73.00–84.50)	83.00 (80.00–95.00)	81.50 (72.00–92.25)	83.50 (81.00–93.25)	84.50 (74.00–96.75)	*H* = 7.084 *p* = 0.214

Data are presented as the median (interquartile range) or number of cases for categorical variables. The *p*-value in the last column was determined using the Kruskal–Wallis *H* test or the Fisher–Freeman–Halton exact test

*CG* control group, *TOP* topical therapy, *MTX* methotrexate, *ADA* adalimumab, *SEC* secukinumab, *GUS* guselkumab, *BMI* body mass index, *PASI* Psoriasis Area Severity Index, *BSA* body surface area, *BP* blood pressure

**Fig. 4:**
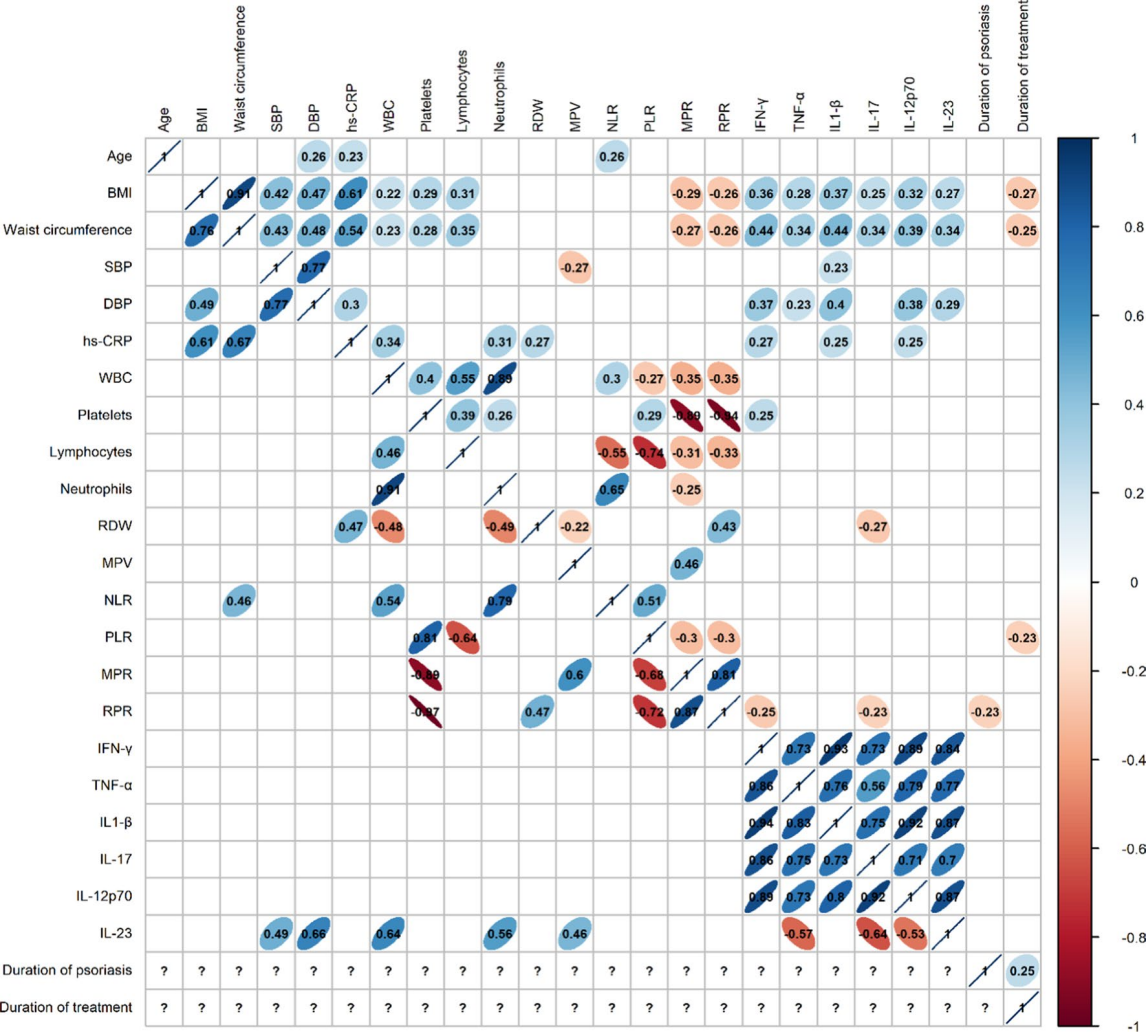
Correlation matrix with Spearman’s correlation coefficients for the control group are shown below the main diagonal and those for the treated patients above the main diagonal. Only significant correlations are shown, others are omitted from the matrix (*α* = 0.05) indicated. BMI, body mass index; SBP, systolic blood pressure; DBP, diastolic blood pressure; hs-CRP, high-sensitivity C-reactive protein; WBC, white blood cells; RDW, red cell distribution width; MPV, mean platelet volume; NLR, neutrophil-to-lymphocyte ratio; PLR, platelet-to-lymphocyte ratio; MPR, median mean platelet volume-to-platelet ratio; RPR, mean RDW-to-platelet ratio; IFN-γ, interferon-γ; TNF, tumor necrosis factor; IL, interleukin; question mark symbol (?) = not applicable

In contrast to the non-specific inflammatory markers, selected disease-specific inflammatory markers, namely IFN-γ, TNF, IL-1β, IL-12p70, and IL-17, were significantly elevated in the five patient groups compared to those in the control group. There were no significant differences in IL-23 levels in the five patient groups compared to controls (Fig. 3). Despite treatment with inhibitors of certain interleukins (adalimumab inhibits TNF, secukinumab inhibits IL-17, and guselkumab inhibits IL-23), we did not observe that the levels of these target cytokines were significantly lower than with other types of treatment. We found that males had significantly higher levels of all six cytokines measured (IFN-γ, TNF, IL-1β, IL-12p70, IL-17, and IL-23) compared to females (Mann–Whitney *U* test, *p* < 0.05). The correlation matrix shows correlations between the inflammatory markers, patient demographics, and anthropometric measurements (Fig. 4). As shown in Fig. 4, BMI and waist circumference were positively correlated with all selected disease-specific inflammatory markers and hs-CRP in patients with psoriasis. We observed no such correlations in the control group. In addition, BMI and waist circumference correlated negatively with the duration of treatment. Selected disease-specific inflammatory markers were also correlated with each other and with diastolic blood pressure.

## Discussion

Our results suggest that despite successful treatment of psoriasis with five different therapies, including three biologics, patients still exhibited a residual inflammatory imprint characterized by elevated disease-specific cytokines compared to control subjects. In contrast, non-specific inflammatory markers showed no significant differences compared to the control group, suggesting that non-specific inflammation decreases and normalizes with successful treatment. The residual disease-specific cytokines correlated with patients’ BMI and waist circumference. These results shed additional light on the treatment-related changes in inflammation and could be clinically relevant.

We investigated systemic inflammation by measuring two types of inflammatory markers (non-specific and disease-specific) in young patients with psoriasis who were successfully treated with five different types of treatment (topical therapy, methotrexate, adalimumab, secukinumab, and guselkumab). Given the significant burden of cardiovascular disease and metabolic disease, to which systemic inflammation can be a major contributor, it is important to know whether successful treatment of psoriasis reduces systemic inflammation and cytokines that are present in both atherosclerosis and psoriasis, such as IL-17 [12]. In addition, although this is only speculation, residual inflammation may contribute to the recurrence or worsening of psoriasis after discontinuation of successful treatment. Overall, residual inflammation is a topic that has not yet been extensively studied, and its clinical relevance remains to be clarified. We studied residual inflammation in a wide range of patients, reflecting the real-life situation in which patients differ in terms of disease severity and duration, as well as treatment modalities. To our knowledge, this is the first study to investigate residual non-specific and disease-specific inflammatory markers in successfully treated young patients with psoriasis, treated with five different treatments at a stable disease stage. We found that disease-specific inflammatory markers were elevated in successfully treated patients. Among several markers, the following were elevated: IFN-γ, TNF, IL-1β, IL-12p70, and IL-17, while only the levels of IL-23 were comparable to those of the control group. The reason for the difference between IL-23 and other disease-specific markers is unclear. Another observation also deserves attention: the residual levels of disease-specific cytokines in patients treated with topical agents and methotrexate were surprisingly similar to those in patients treated with biologics. Regarding the separate groups, we found only a few individual, and not generalized, differences. Except for IL-23, levels of other cytokines targeted with biologics (TNF, IL-17) were also similarly elevated in patients treated with specific antibodies or with another treatment. We found that all disease-specific inflammatory markers correlated with BMI and waist circumference. Increased IL-17 levels in the secukinumab group compared to other patient groups could be due to IL-17 bound to secukinumab, although this was not explicitly measured. In addition, males had slightly higher levels of all six cytokines measured compared to females. We cannot provide an explanation for this result.

There is data on circulating inflammatory markers in psoriasis, but, specifically, residual inflammation in successfully treated patients has not been studied. It is known that inflammatory markers are elevated in psoriasis patients. Some studies investigated the presence of several disease-specific inflammatory cytokines such as TNF [13], IL-1β [7], IL-17A [14, 15], IL-23 [15], and other cytokines in patients with psoriasis, which were elevated compared to controls. A recent systematic review and meta-analysis found that IFN-γ, TNF, IL-2, IL-6, IL-8, IL-18, IL-22, chemerin, lipocalin-2, resistin, sE-selectin, fibrinogen, and C3 were elevated in patients with psoriasis compared to healthy controls, while IL-1β, IL-4, IL10, IL-12, IL-17, IL-21, IL-23, visfatin, and omentin were not significantly different in patients with psoriasis compared to controls [16]. Another recent meta-analysis, which summarized data from several small studies showed that among numerous inflammatory markers, elevated serum concentrations of the inflammatory cytokines IL-2, IL-17, IL-18, and IFN-γ are present in untreated psoriasis patients [17]. Conflicting results have been reported on the serum concentration of IL-17 in psoriasis patients. While some studies found elevated levels [18–20], others found no difference compared to healthy controls [21, 22]. Other studies have shown that disease-specific inflammatory markers correlate with disease severity, which is primarily measured by the PASI [8, 10, 23]. Overall, disease-specific inflammatory markers were mainly determined to investigate the correlation with psoriasis severity and/or as markers of treatment efficacy. Regarding non-specific inflammatory markers, it has been shown that hs-CRP is elevated in psoriasis patients and that hs-CRP serves as a marker for subclinical atherosclerosis in psoriasis patients [24, 25]. Again, the measurements were performed in different patient groups and clinical scenarios than in our case and could not be extrapolated to residual inflammation.

Our results indicate that although non-specific inflammatory markers are effectively reduced by treatment, disease-specific markers persist. The persistence of disease-specific markers may indicate that the skin response to treatment, as measured by the PASI, does not fully reflect the extent of systemic inflammation. On the other hand, this persistent inflammatory imprint could also explain, at least in part, the chronic and recurrent nature of psoriasis. Previous research suggests that the chronic and recurrent nature of psoriasis may be due to local immunologic memory on the skin, driven by tissue-resident memory T cells [26]. Our results suggest that in addition to local immunologic memory, residual disease-specific systemic inflammatory activity may also play a role in disease recurrence. Thus, our results support the prevailing hypothesis that biologic therapy is highly effective but only suppresses the activity of pathogenic immune cells without eradicating them [27]. Our results emphasize the potential role of overweight in residual inflammation. Indeed, disease-specific inflammatory markers correlated with BMI and waist circumference. This could be related to the inflammatory activity of adipose tissue, which secretes pro-inflammatory cytokines such as TNF, IL-1, and IL-6, which could further exacerbate systemic inflammation that overlaps with psoriasis [16, 28]. At this point, we can only speculate about the explanation. However, the clinical value of this observation needs to be investigated in a study examining the role of weight reduction in reducing residual inflammation [29]. In any case, this observation reflects the relationship between psoriasis, systemic inflammation, and excess body fat/weight. There is no doubt that psoriasis and obesity are closely intertwined [16, 28].

We have also investigated novel CBC-derived inflammatory markers that have been shown to be useful cardiovascular risk markers in patients with psoriasis [29–31]. It was shown that NLR and PLR decreased after treatment with TNF inhibitors [32, 33]. These markers did not differ significantly in the patient groups or the control group, suggesting that cardiovascular risk is not increased in successfully treated patients compared to the control group. This could indicate that the persistence of disease-specific inflammatory markers, which are also involved in the pathogenesis of atherosclerosis [29, 30, 34], does not significantly increase the cardiovascular risk after successful treatment. On the other hand, CBC-derived inflammatory markers may not be a suitable tool to assess cardiovascular risk in successfully treated psoriasis patients. Nevertheless, more data are needed to draw definitive conclusions because there are so many factors that influence cardiovascular risk in psoriasis patients, which may even differ in different clinical scenarios and still need to be clarified. Our results also showed that there was an inverse correlation between the duration of treatment and BMI in the psoriasis groups. These results emphasize the need for concurrent treatment of psoriasis and excess body fat/weight.

Our study has several limitations. The lack of previous data on inflammatory markers before initiation of therapy is a limitation of the cross-sectional study. However, we did not focus on the inflammatory changes before and after treatment, but on their levels in the stable phase of the disease. Another limitation was the relatively small number of patients in each group. However, similar results in three groups of patients treated with biologic therapy support the credibility of the results and conclusions.

## Conclusion

Our results suggest that despite successful treatment with topical therapy, methotrexate, adalimumab, secukinumab, or guselkumab, young patients with psoriasis still exhibit a residual systemic inflammatory imprint, characterized by elevated disease-specific inflammatory markers (IFN-γ, TNF, IL-1β, IL-12p70, IL-17, but not IL-23) compared to controls, whereas they do not differ for non-specific inflammatory markers. The residual values of the disease-specific markers were similar regardless of treatment option, and these residual disease-specific inflammatory markers correlated with BMI and waist circumference. The residual disease-specific inflammatory burden may be associated with cardiovascular risk and may reflect the potential for psoriasis recurrence and exacerbation after treatment discontinuation. The effect of weight reduction on residual inflammation should be investigated in further studies.

## Data Availability

The data is added as supplementary material and can be made available on reasonable request.
